# Left Atrial Appendage Occlusion Device Placement Aborted Because of Coronary Artery Bypass Graft

**DOI:** 10.1016/j.jaccas.2023.101740

**Published:** 2023-01-30

**Authors:** John Yin, Garret Hillsdon-Smith, Zachary J. Rhinehart, David Kaczorowski, Sandeep Jain, Harikesh Subramanian

**Affiliations:** a Department of Anesthesiology and Perioperative Medicine, University of Pittsburgh Medical Center, Pittsburgh, Pennsylvania, USA; b Heart and Vascular Institute, University of Pittsburgh Medical Center, Pittsburgh, Pennsylvania, USA; c Department of Cardiothoracic Surgery, University of Pittsburgh Medical Center, Pittsburgh, Pennsylvania, USA

**Keywords:** anesthesia, atrial fibrillation, atrial flutter, echocardiography, imaging, CABG, coronary artery bypass graft, LAA, left atrial appendage, LAAO, left atrial appendage occlusion, SVG, saphenous vein graft, TEE, transesophageal echocardiography

## Abstract

Left atrial appendage occlusion device (LAAO) implantation among patients who have had coronary artery bypass grafting can be challenging. We report a case of scheduled LAAO device implantation that was aborted due to the anomalous course of a bypass graft that appeared to be adherent to the left atrial appendage. (**Level of Difficulty: Intermediate.**)

## Central Illustration

**Figure:**
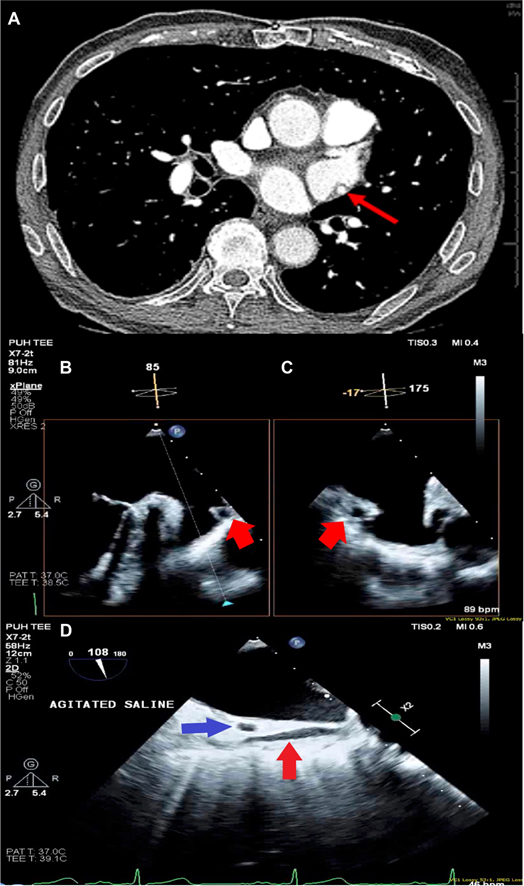

**Figure 1:**
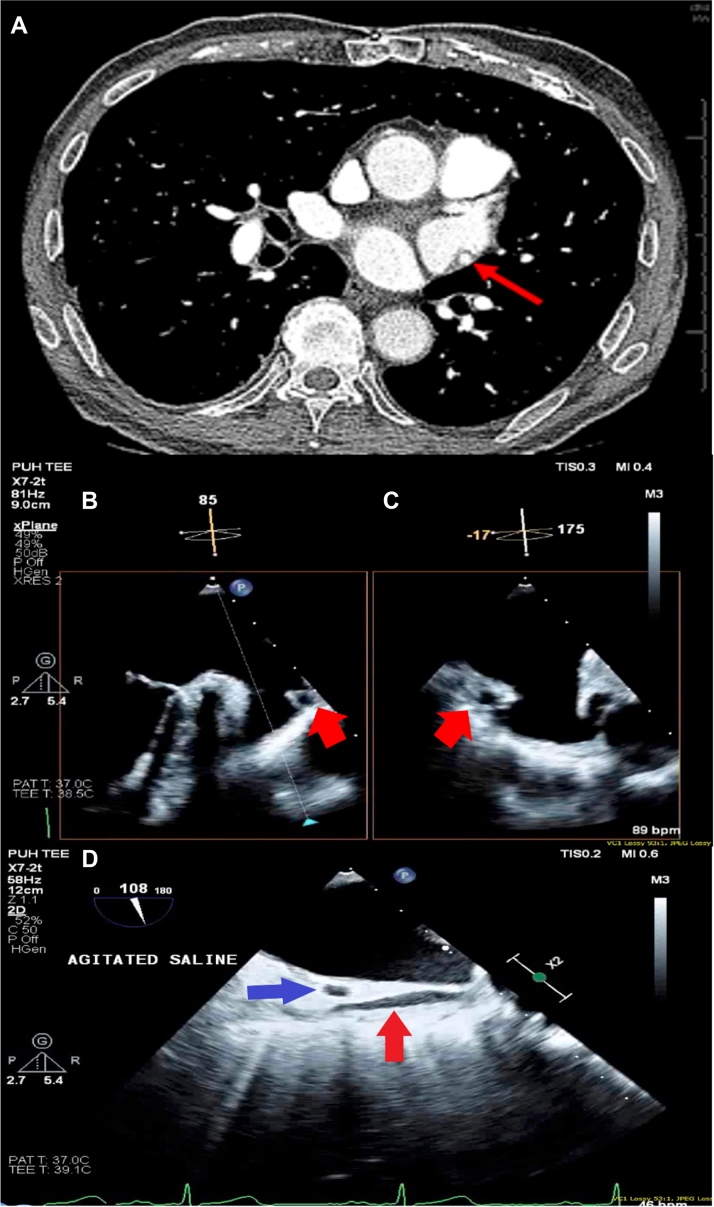
Images of Bypass Graft in 2 Imaging Modalities **(A)** Prior computed tomography angiography showing saphenous vein graft adjacent to left atrial appendage (LAA). Axial slice with **red arrow** pointing to bypass graft. **(B to D)** Left atrial appendage LAA views on transesophageal echocardiography at 85°, –17°, and 108°. LAA, anomalous vessels seen in 3 transducer angles along with cross-section of left circumflex artery. **Blue arrow** = left circumflex artery. **Red arrow** = anomalous vessel.

**Figure 2:**
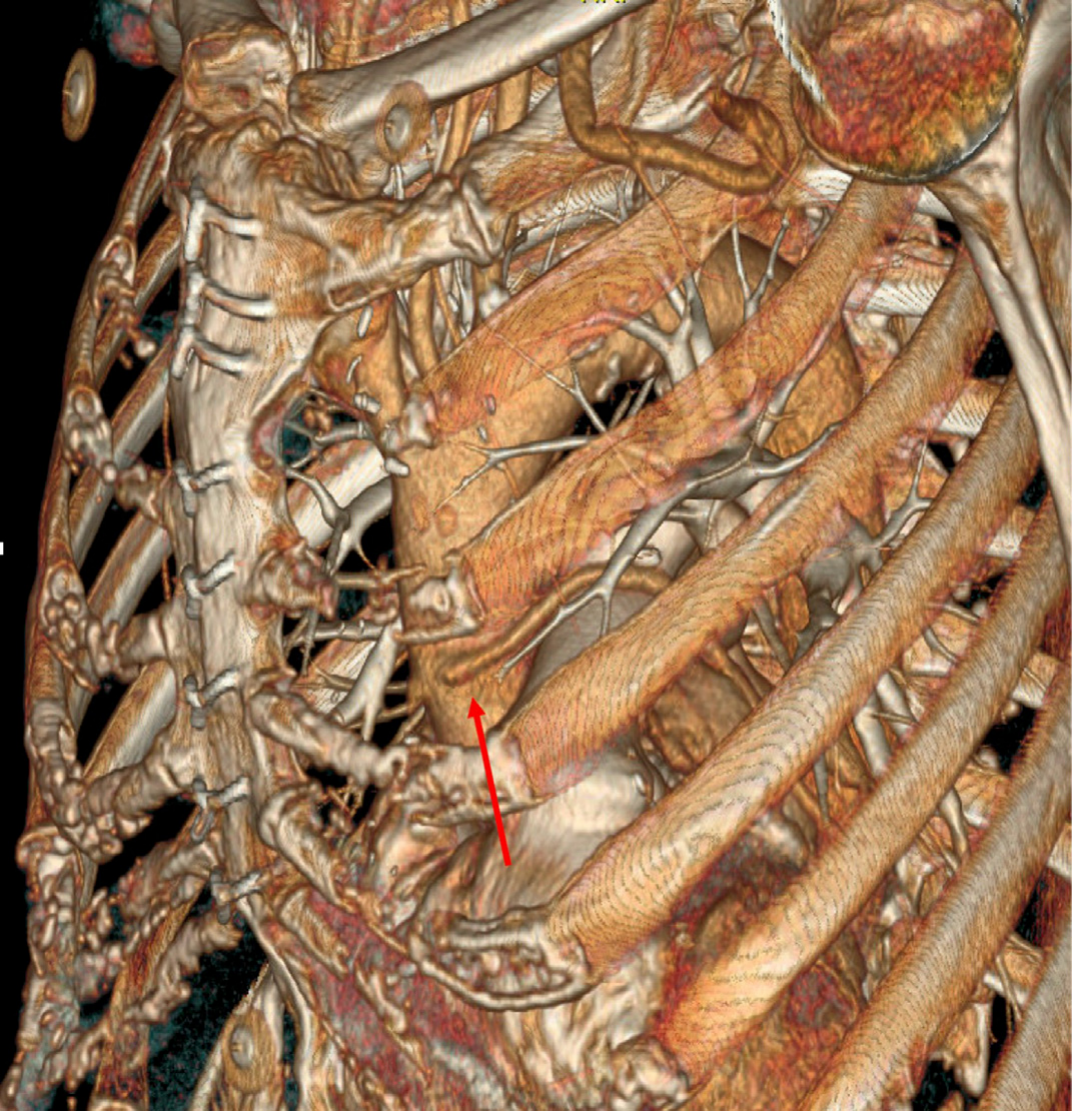
3-Dimensional Rendering of Bypass Graft **Red arrow** points to the origin of the saphenous vein graft bypass graft. The takeoff of the graft is shown coming from the ascending aorta and traversing over the pulmonary artery and overlaying the left atrial appendage.

A 90-year-old man with permanent atrial fibrillation and a CHA_2_DS_2_-VASc score of 4 presented for implantation of a left atrial appendage occlusion (LAAO) device with WATCHMAN FLX. Notable medical history included a 3-vessel coronary artery bypass graft (CABG) 10 years prior (left internal mammary to left anterior descending artery, saphenous vein graft [SVG] to first obtuse marginal and to posterior descending artery) that was complicated by the need for repeated exploration on postoperative day 1 because of bleeding, persistent vertigo, and stage 3 chronic kidney disease with a baseline creatinine of 1.8 mg/dL (estimated glomerular filtration rate 30, chronic kidney disease III). Two years before the scheduled LAAO, he underwent a percutaneous mitral valve repair for severe mitral valve regurgitation with completion of preprocedural and intraprocedural transesophageal echocardiography (TEE) without evidence of left atrial appendage (LAA) abnormalities. Computed tomography angiography before LAAO placement was not ordered, but prior computed tomography angiography for workup of atypical chest pain visualized the bypass grafts (Figures 1A and 2**,** Video 1).

During the LAAO procedure, TEE showed the LAA diameter and depth to be 25 and 31 mm at 0° and 45°, 23 and 29 mm at 90°, and 22 and 30 mm at 135°. No LAA thrombus was seen, but in-depth visualization of the LAA found a structure that appeared to be a vessel crossing adjacent to the LAA near the ostium (Figures 1B and 1C, Video 2). Agitated saline echo contrast material injected did not appear in the vessel (Figure 1D, Video 3), but endocardial border-enhancing agent showed late filling in the vessel, which suggested an arterial origin rather than an anomalous venous connection (Video 4). Given the patient’s CABG history, the vessel was suspected to be 1 of the venous grafts implanted, likely SVG to obtuse marginal. Although the option of performing a coronary catheterization to study the function of the graft was considered, it was deferred, given the patient’s kidney disease and age. Concerned that the deployment of the device proximally would lead to graft vessel compression and subsequent myocardial ischemia, perforation or, tamponade and that distal deployment would lead to incomplete LAA occlusion while risking perforation from device manipulation around the vessel, the procedure was aborted.

Despite current literature demonstrating low rates of complications from LAAO, several case studies have described complications involving cardiac vessels, including ischemia of left circumflex from device compression and pulmonary artery perforation.^1^, ^2^, ^3^ These reports demonstrate the need to be judicious in evaluating the anatomy surrounding the LAA, especially in patients with atrial fibrillation and a history of CABG because grafting can complicate routine LAAO placements by altering the typical appendage anatomy. Grafts to the circumflex may be near the LAA either laterally or coursing between the appendage and myocardium. Our case reinforces the importance of periprocedural imaging such as TEE or cardiac CT in the assessment of LAA anatomy, weighing risks and benefits of LAAO deployment on an individual basis and perhaps avoiding overlaying bypass grafts near LAA if possible, in case LAAO device placements are indicated in the future.

## Funding Support and Author Disclosures

The authors have reported that they have no relationships relevant to the contents of this paper to disclose.

## Appendix

Video 1 Coronal Slices from Computed Tomography Angiography Bypass graft is adhering to left atrial appendage (LAA) appearing to originate from the ascending aorta superiorly and visualized to have a course adherent to the LAA.

Video 2 Transesophageal Echocardiography View of Left Atrial Appendage and Anomalous Vessel at 2 Perpendicular Angles At –17°, the anomalous vessel is seen to the left, and a vessel-like structure, likely trabeculae of the left atrial appendage, is seen to the right.

Video 3 Transesophageal Echocardiography View of Left Atrial Appendage and Anomalous Vessel During Agitated Saline Injection No agitated saline was visualized within the anomalous vessel during saline injection, excluding a venous cause.

Video 4 Transesophageal Echocardiography View of Left Atrial Appendage and Anomalous Vessel During Enhancement Agent Injection Late filling is noted within the vessel.
